# Galectina-3 em Pacientes com Pericardite Constritiva Crônica

**DOI:** 10.36660/abc.20190152

**Published:** 2020-05-12

**Authors:** Fábio Fernandes, Dirceu Thiago Pessoa de Melo, Felix José Alvarez Ramires, Ester Cerdeira Sabino, Carlos Henrique Valente Moreira, Luiz Alberto Benvenutti, Viviane Tiemi Hotta, Ana Luiza Carrari Sayegh, Francis Ribeiro de Souza, Ricardo Ribeiro Dias, Charles Mady

**Affiliations:** 1 HC FM USP São Paulo SP Brasil Universidade de São Paulo Instituto do Coração HC-FMUSP - Unidade Clínica de Miocardiopatias e Doenças da Aorta,São Paulo, SP – Brasil; 2 Universidade de São Paulo Faculdade de Medicina Hospital das Clínicas Instituto do Coração São Paulo SP Brasil Universidade de São PauloUniversidade de São Paulo Faculdade de Medicina Hospital das Clínicas Instituto do Coração, São Paulo, SP – Brasil; 3 Universidade de São Paulo Instituto de Medicina Tropical de São Paulo São Paulo São Paulo SP Brasil Universidade de São PauloUniversidade de São Paulo - Instituto de Medicina Tropical de São Paulo São Paulo, São Paulo, SP – Brasil; 4 Emilio Ribas Institute for Infectious Diseases São Paulo SP Brasil Emilio Ribas Institute for Infectious DiseasesEmilio Ribas Institute for Infectious Diseases, São Paulo, SP – Brasil

**Keywords:** Pericardite Constritiva/cirurgia, Galectina 3, Diferenciação Celular, Pericardiectomia/métodos, Fibrose

## Abstract

**Fundamento:**

A galectina-3 (Gal-3) é uma molécula pró-inflamatória e pró-fibrótica, envolvida na patogênese da insuficiência cardíaca. O papel da Gal-3 em pacientes com pericardite constritiva crônica (PCC) não está claro.

**Objetivo:**

O objetivo deste estudo foi avaliar os níveis de Gal-3 em pacientes com PCC e correlacioná-los com parâmetros clínicos, funcionais e histológicos.

**Métodos:**

Nós avaliamos prospectivamente 25 pacientes sintomáticos com PCC agendados à pericardiectomia e 21 controles sadios. Os pacientes foram submetidos à avaliação clínica, medidas de Gal-3 e peptídeo natriurético do tipo B (BNP), ecocardiografia, ressonância magnética cardíaca e teste cardiopulmonar de exercício (TCPE) no período basal. Seis meses após a pericardiectomia, repetiu-se o TCPE. Um erro alfa < 5% foi considerado estatisticamente significativo, com um intervalo de confiança de 95%.

**Resultados:**

Foram incluídos 25 pacientes com idade mediana de 45 anos. A etiologia foi principalmente idiopática (n = 19, 76%), e 14 (56%) apresentaram classe funcional *New York Heart Association* (NYHA) III/IV. Os valores medianos de BNP e Gal-3 foram 143 (89-209) pg/dL e 14,8 (9,7-17,2) ng/mL, respectivamente. Os níveis de Gal-3 não foram estatisticamente maiores nos pacientes com PCC que em controles (p = 0,22). Não foram encontradas correlações significativas da Gal-3 com BNP, medidas ecocardiográficas e de ressonância magnética cardíaca, e achados histológicos. Após a pericardiectomia, encontrou-se uma correlação estatisticamente significativa entre Gal-3 e medidas do TCPE – duração do teste (r = –0,79; p < 0,001) e tempo de exercício (r = –0,79; p < 0,001).

**Conclusões:**

Pacientes com PCC apresentaram níveis normais de Gal-3, quando comparados aos indivíduos controles. A Gal-3 não se correlacionou com medidas morfológicas e funcionais antes da pericardiectomia. No entanto, associações entre Gal-3 e intolerância ao exercício após pericardiectomia pode sugerir um papel da Gal-3 na predição de prognóstico após a pericardiectomia. (Arq Bras Cardiol. 2020; 114(4):683-689)

## Introdução

Pacientes com pericardite constritiva crônica (PCC) apresentam espessamento do pericárdio que leva à restrição do enchimento diastólico dos ventrículos. Nos estágios iniciais, a apresentação clínica da PCC é geralmente assintomática e inespecífica. Os sintomas podem ser atribuídos à disfunção diastólica biventricular e incluem fadiga e tolerância reduzida ao exercício.^1^

A evolução da inflamação do pericárdio é um evento contínuo. A constrição do pericárdio após pericardite aguda parece estar relacionada à proliferação de fibroblastos e exsudato fibrinoso, resultando em um pericárdio espesso e inelástico.^2^ Contudo, os mecanismos que levam à fibrose e calcificação do pericárdio na PCC ainda são pouco conhecidos.

A galectina-3 (Gal-3), uma lecitina que se liga à beta-galactosidase, é secretada por macrófagos ativados e está envolvida no processo de fibrogênese. A Gal-3 é também um forte mediador pró-inflamatório.^3 , 4^

Dados sobre os níveis de galectina em pacientes com doença do pericárdio são limitados. Em um estudo piloto, Ntsekhe et al.,^5^ estudaram pacientes com pericárdio normal em pacientes com pericardite tuberculosa para definir níveis de Ac-SDKP (N-acetil-seril-aspartil-lisil-prolina) e Gal-3 em fluidos de pericárdio normal. Os autores encontraram que o AcSDKP, um tetrapeptídeo com propriedades antifibróticas, e a Gal-3 são detectáveis em fluidos de pericárdio normal, e que a pericardite tuberculosa associou-se com níveis baixos de AcSDKP no pericárdio e níveis normais de Gal-3. Contudo, ainda não está claro o papel da Gal-3 em pacientes com PCC.

Dado o papel da inflamação do pericárdio e da fibrose na patogênese na PCC, nossa hipótese é a de que a Gal-3 pode servir como um biomarcador ou modulador de gravidade da pericardite constritiva. O objetivo deste estudo foi avaliar os níveis plasmáticos de Gal-3 em pacientes com PCC e correlacionar esses níveis com parâmetros funcionais e histológicos.

## Métodos

### População do estudo

**Figura 1:**
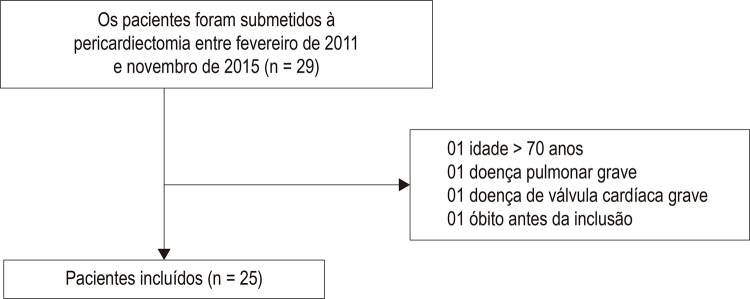
– processo de rastreamento.

Neste estudo prospectivo, foram incluídos 33 pacientes com pericardite constritiva comprovada cirurgicamente. Vinte e nove pacientes foram submetidos à pericardiectomia radical de fevereiro de 2011 a novembro de 2015 em um hospital terciário em São Paulo, Brasil. Quatro pacientes foram excluídos do estudo pelos seguintes critérios de exclusão: idade superior a 70 anos, doença pulmonar grave de acordo com teste pulmonar, e doença de válvula cardíaca moderada/grave. Vinte e cinco pacientes foram comparados com 21 indivíduos sadios, sedentários, sem doença cardíaca (grupo controle). O tamanho da amostra foi definido por conveniência. Assumiu-se o diagnóstico de PCC com base em critérios clínicos, ecocardiográficos, e de imagem de RMC, seguindo-se as diretrizes da *European Society of Cardiology* , confirmado por cirurgia.^1^ Os seguintes procedimentos foram realizados durante a internação para a cirurgia: níveis séricos de peptídeo natriurético do tipo B (BNP), ecocardiografia transtorácica, teste cardiopulmonar de exercício e RMC ( Figura 1 ).

A constrição tuberculosa foi definida por biópsia do pericárdio, pela presença de granuloma caseosa, ou quando a reação em cadeia da polimerase foi positiva para M *ycobacterium sp* . A constrição pós-cirúrgica foi definida como pericardite constritiva após a cirurgia cardíaca. Constrição secundária à doença inflamatória sistêmica foi definida em dois pacientes com lúpus eritematoso sistêmico. Constrição idiopática foi definida para pacientes que não se qualificaram em nenhum dos grupos anteriores.

### Delineamento do estudo

Este foi um estudo caso controle com indivíduos controles (sadios) pareados por idade e sexo.

### Procedimentos

#### Procedimento de pericardiectomia

Foi realizada esternotomia mediana em todos os casos, sem *bypass* cardiopulmonar. Pericardiectomia total foi realizada com excisão do pericárdio anteriormente, estendendo-se aos nervos periféricos e pericárdio diafragmático. Quando esse procedimento era tecnicamente viável, tentou-se a remoção do pericárdio visceral e parietal.

#### Teste cardiopulmonar de exercício

A capacidade funcional foi avaliada pelo TCPE seguindo-se as diretrizes da *American Heart Association* .^6^ A avaliação foi realizada em uma esteira (Ergoline – Via Sprint 150 P), usando-se protocolo modificado de Balke, e velocidade variando de 2 a 3,4 mph, e aumento de inclinação de 2% por minuto. Os pacientes foram posicionados na esteira e conectados a um transdutor volumétrico usando um clip nasal. Em seguida, realizou-se monitoramento eletrocardiográfico ( *Micromed - Cardio PC 13* ). As frações de oxigênio (O_2_) e de dióxido de carbono (CO_2_) foram medidas em cada ciclo respiratório. A avaliação foi realizada utilizando-se um sistema computadorizado ( *Sensormedics, Vmax Analyzer Assembly, Encore 29S* ). A pressão arterial foi medida pelo método auscultatório, a cada dois minutos de exercício. No período de recuperação, a pressão arterial foi medida no minuto um, dois, quatro e seis. O TCPE foi considerado máximo quando o indivíduo alcançou pelo menos um dos seguintes parâmetros: razão de troca respiratória > 1,10; frequência cardíaca > 95% do previsto para a idade, e cansaço extremo.

Os níveis de BNP foram determinados utilizando-se o kit ADVIA Centaur® (Siemens Medical Solutions Diagnostic, Los Angeles, California, USA), e as amostras processadas em equipamento automatizado da mesma marca, em até duas horas, conforme recomendado pelo fabricante.

Os níveis de Gal-3 foram determinados pelo ensaio imunoenzimático por fluorescência (Enzyme-Linked Fluorescent Assay, ELFA), e medidos usando o Biomerieux Vidas 30 (Biomerieux, Marcy l’Etoile, LY-França). A calibração do teste foi realizada seguindo-se recomendações do fabricante.

#### Ecocardiografia

O estudo de ecocardiografia foi realizado utilizando-se o aparelho de ultrassom Sequoia 512 (Acuson, Mountain View, California, USA) com um transdutor de 2.5 MHz. Todas as medidas foram realizadas seguindo-se as recomendações da *American Society of Echocardiography* .^7^ Um respirômetro nasal foi usado para registro simultâneo da respiração. O teste foi realizado por um examinador, cego para outras avaliações do protocolo. Exame bidimensional foi realizado a partir das janelas paraesternal, apical e subcostal. Imagens dos cortes paraesternal e apical, bem como registros do modo M foram usadas para detectar movimentação do septo ventricular. Cortes apicais também foram utilizados para detectar distorção do contorno ventricular causada por constrição do pericárdio. O corte subcostal foi usado par identificar diâmetros da veia cava inferior. Dados de Doppler foi obtido das janelas subcostal, supraventricular direito e paraesternal. Da janela apical, registros de Doppler de onda pulsada nas extremidades do folheto mitral foram usados para medir a velocidade diastólica precoce (E) e atrial (A), tempo de desaceleração da onda E, e variação respiratória na velocidade E. A avaliação por Doppler tecidual da movimentação do anel mitral foi usada para registrar e comparar a velocidade diastólica precoce no anel mitral lateral e septal.

## Análise estatística

Foi realizada análise descritiva dos dados. Para os dados quantitativos, medidas de tendência central e dispersão foram descritas em mediana e intervalos interquartis. Os dados qualitativos foram descritos em frequência e porcentagem. Os níveis de Gal-3 foram comparados entre controles e grupos caso utilizando o teste de soma de postos de Wilcoxon, e o teste do qui-quadrado ou o teste exato de Fisher usado para dados categóricos. A correlação de Spearman foi usada para avaliar a associação entre os níveis de Gal-3, e parâmetros de ecocardiografia, RMC e teste ergométrico. Um erro alfa < 5% foi considerado estatisticamente significativo, com intervalo de confiança de 95%.

## Aspectos éticos

O comitê de ética da instituição aprovou este estudo, o qual foi realizado de acordo com a Declaração de Helsinki. O comitê de ética local aprovou o protocolo do estudo, e todos os participantes assinaram o termo de consentimento.

## Resultados

### Características basais

Vinte e cinco pacientes com pericardite constritiva foram submetidos à pericardiectomia. A idade mediana foi 45 anos (33-57), com predominância de homens (n = 19, 76%). O índice de massa corporal (IMC) mediano foi 25,6 kg/m^2^. As comorbidades foram hipertensão, tabagismo, diabetes tipo 2, e doença arterial crônica. Todas as características basais encontram-se na Tabela 1 . No grupo controle, a idade mediana foi 44 (33-53) anos, e 19 eram homens.

**Tabela 1:** – Parâmetros clínicos e laboratoriais

Características	Medidas
Sexo masculino, n (%)	19 (76)
Idade (anos), mediana (IIQ)	45 (33-57)
IMC, kg/m^2^, mediana (IIQ)	25,6 (22-27)
Duração dos sintomas (meses), mediana (IIQ)	24 (12-36)
Tempo de hospitalização, mediana (IIQ)	8 (7-16)
Tempo na UTI após o procedimento, mediana (IIQ)	2 (2-3)
**Comorbidades, n (%)**	
Hipertensão	4 (16)
Diabetes tipo 2	2 (8)
Doença arterial crônica	3 (12)
Tabagismo	5 (20)
Fibrilação atrial	10 (40)
Baixa voltagem ECG	6 (24)
Calcificação (Raio X)	11 (44)
Derrame pleural (Raio X)	5 (20)
**Classe funcional NYHA, n (%)**	
I	4 (16)
II	7 (28)
III	11 (44)
IV	3 (12)
**Sinais clínicos, n (%)**	
Estase jugular	22 (88)
Edema	22 (88)
Ascite	18 (72)
Hepatomegalia	16 (64)
*Knock* pericárdico	12 (48)
Sinal de Kussmaul	6 (24)
Pulso paradoxal	5 (20)
**Medidas laboratoriais– mediana (IIQ)**	
Galectina-3, ng/mL*	14,8 (9,7-17,2)
Hemoglobina, g/dL,	13,4 (12,8-14,3)
Creatinina, mg/dL,	1,02 (0,99-1,26)
PCR, mg/dL	5,4 (3,2-9,4)
BNP, pg/mL	143 (89-209)

Variáveis contínuas apresentadas em mediana e intervalo interquartil (IIQ). Dados categóricos estão apresentados em porcentagem. *risco de morte no pós-operatório calculado pelo EuroSCORE (%). IMC: índice de massa corporal; ECG: eletrocardiograma; UTI: unidade de terapia intensiva; NYHA: New York Heart Association; BNP: peptídeo natriurético do tipo B; PCR: proteína C-reativa

Em relação às características clínicas, a duração (mediana) dos sintomas antes da internação foi 24 meses (12-36). O período de internação hospitalar, em mediana, foi de oito dias, e de internação na unidade de terapia intensiva (UTI) foi de dois dias. Os principais sinais clínicos observados dos pacientes estiveram relacionados com insuficiência cardíaca direita, distensão da veia jugular (n = 22, 88%), edema (n = 22, 88%), ascite (n = 18,72%) – e 16 (64%) apresentaram hepatomegalia ao exame físico ( Tabela 1 ).

Quatorze (56%) pacientes apresentaram classe funcional III/IV da *New York Heart Association* (NYHA) na admissão. Ao analisar os sinais clínicos, pacientes com ascite apresentaram níveis maiores de Gal-3 [16,2 ng/mL (11,6-17,5)] em comparação àqueles sem ascite [8,2 ng/mL (6,6-14,8)], porém sem diferença estatisticamente significativa. Não foi observada associação dos níveis de Gal-3 com nenhum dos sinais descritos.

O diagnóstico etiológico mais frequente foi idiopático (n = 19, 76%), tuberculose (n = 3, 12%), colagenases (n =2, 8%), e pós-cirurgia (n = 1, 4%). Não houve óbito decorrente da pericardiectomia.

### Testes laboratoriais e complementares

Os valores medianos da concentração de hemoglobina, creatinina, proteína C-reativa, BNP e Gal-3 estão apresentados na Tabela 1 . Os níveis de Gal-3 não foram significativamente mais altos nos pacientes com PCC em comparação aos pacientes controles. Os níveis medianos de Gal-3 foram 14,8 (9,7-17,2) ng/mL e 11,8 (10,6-14,2) ng/mL para pacientes com PCC e controles, respectivamente (p = 0,22). Além disso, não foi observada associação significativa entre os níveis de Gal-3 e medidas de ecocardiografia (diâmetro diastólico do ventrículo direito, DDVD; diâmetro sistólico do ventrículo direito, DDVD; diâmetro do átrio esquerdo, fração de ejeção do ventrículo esquerdo) ou medidas da RMC (pressão sistólica da artéria pulmonar, PSAP > 55 mmHg; diâmetro do átrio esquerdo; movimento anormal do septo; dilatação da veia cava e realce tardio do miocárdio e do pericárdio).

### Exames de imagens

Todos os indivíduos foram submetidos à ecocardiografia e à RMC. Os valores medianos de fração de ejeção medida por ecocardiografia e RMC foram 60% e 57%, respectivamente. Ao analisar os parâmetros ecocardiográficos, somente 13 (52%) dos resultados sugeriram pericardite constritiva como diagnóstico. Além disso, espessamento do pericárdio foi observado em 17 (68%) indivíduos, e variação inspiratória no fluxo mitral e tricúspide em 13 (52%), sugerindo restrição diastólica ( Tabela 2 ).

**Tabela 2:** – Variáveis da ecocardiografia e ressonância magnética cardíaca

Ecocardiografia	N	%
Imagem sugestiva de PPC	13	52
Seio aórtico (mm)	30	(29-34)
Diâmetro diastólico do átrio esquerdo (mm)	43.5	(40-47)
Septo interventricular (mm)	8	(8-9)
Parede posterior (mm)	8	(8-9)
DDVD basal (mm)	28	(26-32)
DDVE (mm)	45	(41-46)
DSVE (mm)	29	(27-32)
FEVE (%)	60	(59-66)
Espessamento do pericárdio (> 4 mm)	17	68
Variações do fluxo respiratório (%)	13	52
**Ressonância magnética cardíaca**		
Imagem sugestiva de PCC	23	92
Realce do pericárdio	6	24
Realce do miocárdio	2	8
Movimento anormal do septo	23	92
Aumento do átrio esquerdo	22	88
Dilatação da veia cava	23	92
FEVE (%)	57	(54-62)
Espessura do pericárdio (mm)	6	(5-8)
Espessura do pericárdio (> 4 mm)	21	84

*Variáveis contínuas apresentadas em mediana e intervalo interquartil (IIQ), dados categóricos como porcentagem. DDVD: diâmetro diastólico do ventrículo direito; DDVE: diâmetro diastólico do ventrículo esquerdo; DSVE: diâmetro sistólico do ventrículo esquerdo; FEVE: fração de ejeção do ventrículo esquerdo; PCC: pericardite constritiva crônica.*

As imagens da RMC sugeriram diagnóstico de PCC em 23 (92%) indivíduos. Espessamento (>4 mm) foi encontrado em 21 indivíduos, e as anormalidades mais frequentes foram movimentação anormal do septo e dilatação da aorta e veia cava, ambos observados em 23 (92%) pacientes, seguido de aumento do átrio esquerdo em 22 (88%) ( Tabela 3 ).

**Tabela 3:** – Efeito da pericardiectomia na capacidade funcional

Variáveis	Pré	Pós	p
Velocidade (mph)	2,5 [2-2,5]	3 [2,5-3,3]	0,001
Tempo de exercício (min)	9,5 [6,9-11,7]	9,9 [4,5-14]	0,397
Pico de FC (bpm)	139 [114-160]	159 [138-178]	0,020
VO_2_ no LA (mL/kg/min)	13,5 [11,2-14,6]	16,4 [13,8-20,75]	0,002
LA (%)*	73 [60-81]	69,5 [62,5-78,5]	0,856
VO_2_ (mL/kg/min) pico	18,5 [14,6-22,9]	25,4 [22,3-28,6]	< 0,001
VO_2_ (%) pico*	63 [49,5-70,5]	82 [69,5-95]	< 0,001
V_E_ (L/min) pico	48 [41,3-57,6]	61,7 [44,5-79,9]	< 0,001
V_E_/VCO_2_ RER	35,5 [30-40] 1,1	29 [28-31,5] 1,1	< 0,001 > 0,05

* Porcentagem em relação ao previsto para idade e sexo. Varáveis contínuas são apresentadas em mediana e intervalo interquartil (IIQ), e dados categóricos apresentados em porcentagem. VO_2_: consumo de oxigênio; LA: limiar anaeróbico; FC: frequência cardíaca; VE: ventilação pulmonar; RER: razão de troca respiratória.

Teste cardiopulmonar de exercício

Em todos os indivíduos, o TCPE mostrou-se seguro, sem sérias complicações. Os testes foram considerados efetivos, uma vez que a razão (mediana) de troca respiratória foi 1,1 em ambos os momentos do estudo. De modo geral, após a intervenção cirúrgica, os pacientes apresentaram melhora na capacidade cardiopulmonar ( Tabela 3 ) em termos de velocidade na esteira, pico de frequência cardíaca, pico de consumo de oxigênio (VO_2_ pico) no limiar anaeróbico (LA), LA, e VO_2_ e inclinação V_E_/VCO_2_.

Os valores de BNP não tiveram correlação com parâmetros do TCPE antes ou após o procedimento de pericardiectomia. No entanto, apesar de não termos observado correlação do marcador Gal-3 com parâmetros do TCPE antes da pericardiectomia, observamos uma correlação moderada inversa com duração do teste (r = –0,79; p < 0,001), tempo de exercício (r = -0,79; p < 0,001) e frequência cardíaca no LA (r = 0,60; p = 0,01).

### Estudo histopatológico

A análise histológica foi realizada com 21 amostras. Fibrose grave e calcificação foram achados comuns, encontrados em 19 (90,5%) e 12 (57,1%) das amostras, respectivamente. Inflamação leve foi detectada em 16 casos (76,2%) pelo exame histopatológico. Não foi observada associação estatisticamente significativa entre Gal-3 e esses achados ( Tabela 4 ).

**Tabela 4:** – Análise histopatológica e galectina-3

	Leve	Grave
**Fibrose**		
n (%)	2 (9,5)	19 (90,5)
Gal-3	19,7 [14,2-25,2]	14,8 [9,4-17,2]
**Calcificação**		
n (%)	9 (42,9)	12 (57,1)
Gal-3	16,1 [11,9-20,3]	14,1 [9,5-16,2]
**Inflamação**		
n (%)	16 (76,2)	5 (23,8)
Gal-3	14,5 [10,5-16,9]	16,1 [9,3-25,2]

*Variáveis contínuas apresentadas como mediana e intervalo interquartil. Dados categóricos apresentados como porcentagem. Não foi observada diferença estatisticamente significativa entre os grupos.*

## Discussão

Nosso estudo mostrou que pacientes com diagnóstico de PCC apresentaram níveis normais de Gal-3 no período pré-operatório, comparáveis aos indivíduos controles. Ainda, não observamos correlação significativa de Gal-3 com medidas ecocardiográficas e de RMC ou BNP. Após pericardiectomia, observamos uma melhora no VO_2_ pico e na inclinação V_E_/VCO_2_, os quais são marcadores de um pior prognóstico. Contudo, observamos associações negativas entre Gal-3 e parâmetros de TCPE como a duração do teste e o tempo de exercício.

Gal-3 é conhecido por ser um mediador de fibrose em muitos órgãos, incluindo coração, rim, pâncreas, fígado e pulmão.^8^ No entanto, não foram realizados estudos sobre dados clínicos de pacientes com PCC, associando níveis de Gal-3 com estrutura, função e status funcional do pericárdio.

Ntsekhe et al.,^5^ estudaram os níveis de galectina e AcSDKP em fluidos normais do pericárdio e no derrame pericárdico na tuberculose.^5^ O AcSDKP exerce seu efeito antifibrótico pela inibição da Gal-3, a qual é inativa pela enzima conversora de angiotensina (ECA). Os autores concluíram que níveis diminuídos de AcSDKP, combinados com níveis baixos ou normais de Gal-3 no pericárdio pode explicar a alta incidência de pericardite constritiva associada à pericardite tuberculosa.

A pericardite constritiva é uma doença heterogênea, e o risco de constrição após um episódio agudo está correlacionado com a etiologia. Imazio et al.,^9^ relataram uma incidência de pericardite constritiva < 0,5% em pericardite idiopática ou pericardite viral aguda; 2,8% em doença do tecido conjuntivo; 4,0% para pericardite neoplásica; 20% para pericardite tuberculosa; e 33% para pericardite purulenta. Em nossa coorte, a maioria dos pacientes (76%) apresentaram etiologia idiopática, e não podemos extrapolar nossos resultados a outras etiologias. Somente um estudo com mais pacientes e diferentes etiologias poderia elucidar se a galectina pode modular constrição.

A inflamação é um processo fisiológico que desencadeia fibrose e regeneração tecidual após a lesão. O pericárdio é uma estrutura pouco vascularizada, com fibras compostas de colágeno que geralmente não revela o realce tardio observado com injeção de gadolínio. Em casos de hiperemia e inflamação no pericárdio, ocorre aumento da vascularização e consequente aumento no realce tardio na RMC.^10^ Zurik et al.,^2^ também observaram que realce tardio aumentado do pericárdio na RMC é comum em pacientes com PCC, e está associado com marcadores histológicos de inflamação crônica e neovascularização, o que indicam presença de reação inflamatória dinâmica ativa.^2^ Pacientes com PCC sem aumento de realce tardio do pericárdio apresentaram mais fibrose e calcificação do pericárdio e menos espessamento do pericárdio. Também não observamos nenhuma associação significativa dos níveis de Gal-3 com espessamento do pericárdio e realce tardio do pericárdio avaliado por RMC.

Uma explicação para o motivo pelo qual alguns pacientes com PCC não melhoram após a cirurgia é atrofia do miocárdio após constrição prolongada, constrição residual, ou um processo concomitante no miocárdio que leva à insuficiência cardíaca prolongada apesar de um procedimento de pericardiectomia bem sucedido.^11 , 12^ Outra possibilidade é fibrose do miocárdio. Provavelmente, a fibrose induzida pela Gal-3 é restrita ao miocárdio e não ao pericárdio. Nós também não observamos um aumento nos níveis de galectina e no realce tardio do miocárdio avaliado por RMC e histologia.

O exame cardiopulmonar é a ferramenta mais útil na avaliação objetiva da capacidade de exercício de pacientes com insuficiência cardíaca sistólica e diastólica.^6^ O teste permite avaliar o prognóstico, a eficácia do tratamento, e a escolha pelo transplante cardíaco. Além disso, o TCPE exerce um papel importante na prescrição de exercícios e programas de reabilitação.

Nossos pacientes apresentaram melhora de VO_2_ pico e V_E_/VCO_2_, os quais são dois preditores independentes de mortalidade em pacientes com insuficiência cardíaca com disfunção sistólica ou diastólica.^13 , 14^ Ainda, um aumento no VO_2_ pico está associado com menor ocorrência de reinternações em pacientes com insuficiência cardíaca, demonstrando a importância da pericardiectomia para essa população.^15^

Por outro lado, apesar de muitos estudos terem avaliado o impacto da pericardiectomia na classe funcional dos pacientes com PCC, a maioria desses estudos foram retrospectivos de série de casos.^10 , 12^ Ainda, a avaliação clínica baseada na classificação do NYHA é imprecisa e subjetiva. Alguns pacientes não recuperam a capacidade funcional e a classe funcional NYHA.

As associações entre os níveis de Gal-3 e intolerância ao exercício após a pericardiectomia sugerem o possível papel da Gal-3 na fisiopatologia da pericardite constritiva. Essa hipótese deve ser testada em estudos de acompanhamentos mais longos. O achado de que a Gal-3 é um preditor de melhora da capacidade funcional é relevante, uma vez que sugere que os benefícios da pericardiectomia são menores nos pacientes com níveis mais elevados de Gal-3.

## Conclusão

Os níveis de Gal-3 foram normais nos pacientes com PCC e não se correlacionaram com medidas morfológicas e funcionais. As associações entre os níveis de Gal-3 e intolerância ao exercício após a pericardiectomia sugerem o possível papel da Gal-3 na predição do prognóstico após a pericardiectomia.

### Limitações

A amostra foi composta de pacientes jovens, com predominância de etiologia idiopática, atendidos em um centro de cardiologia terciário. Tal fato pode representar um viés de seleção e limita a validade externa dos resultados. Nós obtivemos somente uma medida da Gal-3 e, por isso, não avaliamos as mudanças dinâmicas nesse biomarcador ao longo do tempo.
