# Supplementary material for: Galectina-3 em Pacientes com Pericardite Constritiva Crônica
Source: Arq Bras Cardiol. 2020 May 12;114(4):683–9. [Article in Portuguese] doi: 10.36660/abc.20190152 (PMC9744340; doi:10.36660/abc.20190152)
Supplement: Translation

# Translation: Galectin-3 Levels in Patients with Chronic Constrictive Pericarditis

**Authors:** Fábio Fernandes, Dirceu Thiago Pessoa de Melo, Felix José Alvarez Ramires, Ester Cerdeira Sabino, Carlos Henrique Valente Moreira, Luiz Alberto Benvenutti, Viviane Tiemi Hotta, Ana Luiza Carrari Sayegh, Francis Ribeiro de Souza, Ricardo Ribeiro Dias, Charles Mady

**Affiliations:** 1 Brazil Instituto do Coração HC-FMUSP - Unidade Clínica de Miocardiopatias e Doenças da Aorta, São Paulo, SP – Brazil; 2 Brazil Universidade de São Paulo Faculdade de Medicina Hospital das Clínicas Instituto do Coração, São Paulo, SP – Brazil; 3 Brazil Universidade de São Paulo - Instituto de Medicina Tropical de São Paulo São Paulo, São Paulo, SP – Brazil; 4 Brazil Emilio Ribas Institute for Infectious Diseases, São Paulo, SP – Brazil

**Keywords:** Pericardite Constrictive/surgery, Galectin 3, Cell Diferentiation, Pericardiectomy/methods, Fibrosis

## Abstract

**Background:**

Galectin-3 (Gal-3) is a proinflammatory, profibrotic molecule implicated in the pathogenesis of heart failure. The role of Gal-3 in patients with chronic constrictive pericarditis (CCP) is not clear.

**Objective:**

The aim of this study was to assess plasma Gal-3 in patients with CCP and correlate it with clinical, functional and histologic parameters.

**Methods:**

We prospectively evaluated 25 symptomatic patients with CCP referred for pericardiectomy and 21 healthy controls. Patients underwent clinical assessment, Gal-3 and B-type natriuretic peptide (BNP) measurements, echocardiography, cardiac magnetic resonance imaging and cardiopulmonary exercise test (CPET) at baseline. Six months after pericardiectomy CPET was repeated. An alpha error < 5% was considered statistically significant, with a confidence interval of 95%.

**Results:**

Twenty-five patients with a median age of 45 years were included. Etiology was mainly idiopathic (n = 19, 76%); and 14 (56%) patients had NYHA functional class III/IV. Median BNP and Gal-3 were 143 (89-209) pg/dL and 14.8 (9.7-17.2) ng/mL, respectively. Gal-3 levels were not significantly higher in CCP patients than in control (p = 0.22). There were no significant correlations of Gal-3 with BNP, echocardiographic and cardiac magnetic resonance measures and histological findings. After pericardiectomy, it was found a statistically significant correlation between Gal-3 and the CPTE measures test duration (r = –0.79; p < 0.001) and exercise time (r = –0.79; p < 0.001).

**Conclusions:**

Patients with CCP had normal levels of Gal-3 as compared to the controls. Gal-3 did not correlate with morphological and functional measures before pericardiectomy. However, the associations between Gal-3 and exercise intolerance after pericardiectomy may suggest a role of Gal-3 in prognosis prediction after pericardiectomy. (Arq Bras Cardiol. 2020; 114(4):683-689)

## Introduction

Patients with chronic constrictive pericarditis (CCP) have pericardial thickness that leads to restriction of diastolic filling of the ventricles. Clinical presentation of CCP is usually indolent and nonspecific in the early stages. Symptoms are attributable to biventricular diastolic dysfunction and include fatigue and decreased exercise tolerance.^1^

The progression of pericardial inflammation is a continuous event. Pericardial constriction after acute pericarditis seems to be related to fibroblast proliferation and fibrinous exudate, resulting in a thickened and inelastic pericardium.^2^ However, the mechanisms underlying pericardial fibrosis and calcification in CCP remain poorly understood.

Galectin-3 (Gal-3), a beta-galactosidase binding lectin, is secreted by activated macrophages and is involved in the fibrogenesis process. Gal-3 is also a strong proinflammatory mediator.^3 , 4^

Limited data are available on galectin levels in patients with pericardial disease. In a pilot study, Ntsekhe et al.^5^ studied patients with normal pericardium and patients with tuberculous pericarditis to define the levels of endogenous Ac-SDKP (N-acetyl-seryl-aspartyl-lysyl-proline) and Gal-3 in normal pericardial fluid. They found that AcSDKP, a tetrapeptide with antifibrotic properties, and Gal-3 are detectable in normal pericardial fluid, and that tuberculous pericarditis was associated with low levels of pericardial AcSDKP and normal Gal-3 levels. Nevertheless, the role of Gal-3 in patients with CPP is not clear.

Given the role of pericardial inflammation and fibrosis in the pathogenesis of CCP, we hypothesized that Gal-3 may serve as a biomarker or modulator of severity in patients with constrictive pericarditis. The aim of this study was to assess plasma Gal-3 levels in patients with CCP and correlate these levels with functional and histologic parameters.

## Methods

### Study population

In this prospective study, 33 patients with surgically proven constrictive pericarditis were included. Twenty-nine patients underwent radical pericardiectomy from February 2011 through November 2015 in a tertiary hospital in São Paulo, Brazil. Four patients were excluded from the study by the following exclusion criteria: patients older than 70 years old, severe pulmonary disease according to a lung test, and moderate/severe valvular heart disease. Twenty-five patients were compared with 21 healthy, physically inactive individuals without heart disease (control group). The sample size was defined by convenience. The presumptive diagnosis of CCP was based on clinical, echocardiographic, and cardiac magnetic resonance (CMR) imaging criteria according to European Society of Cardiology guidelines and proven by surgery.^1^ The following procedures were performed during hospitalization for surgery: measurement of serum B-type natriuretic peptide (BNP) levels, transthoracic echocardiography, cardiopulmonary exercise test (CPET) and CMR imaging test ( Figure 1 depicts the screening process).

**Figure 1:** – Screening process.

Tuberculosis constriction was defined by pericardial biopsy, when caseating granuloma was demonstrated or polymerase chain reaction was positive for *Mycobacterium sp* . Postsurgical constriction was defined as constrictive pericarditis after cardiac surgery. Constriction secondary to systemic inflammatory disease was defined in two patients with lupus erythematosus. Idiopathic constriction was defined when patients did not qualify for any of the previous groups.

### Study design

This was a case control study with controls (healthy subjects) paired by age and sex.

### Procedures

#### Pericardiectomy procedure

Median sternotomy was performed in all cases without cardiopulmonary bypass. Total pericardiectomy was performed with excision of the pericardium anteriorly, extending to both phrenic nerves and diaphragmatic pericardium. When this procedure was technically impossible, removal of parietal and visceral pericardium was attempted.

#### Cardiopulmonary exercise test

Functional capacity was evaluated using the CPET, according to American Heart Association guidelines.^6^ The evaluation was performed on a treadmill ( *Ergoline - Via Sprint 150 P* ) with modified Balke protocol with velocity varying from 2 to 3.4 mph and a ramp increment of 2% per minute. After placement on the treadmill, patients were connected to a volume transducer, with nose clipped, electrocardiographic monitoring ( *Micromed - Cardio PC 13* ) was performed. The oxygen (O_2_) and carbon dioxide (CO_2_) fractions were measured at each respiratory cycle. This evaluation was performed using a computerized system ( *Sensormedics, Vmax Analyzer Assembly, Encore 29S* ). Blood pressure was assessed by the auscultatory method, and measurements were taken every two minutes of exercise. In the recovery period, blood pressure was measured in the first, second, fourth, and sixth minutes. The cardiopulmonary exercise test was considered maximum when the individual reached at least one of the following parameters: respiratory exchange ratio > 1.10, heart rate > 95% of predicted for age, and extreme tiredness.

BNP assays were performed using the ADVIA Centaur® Kit ( *Siemens Medical Solutions Diagnostic, Los Angeles, California, USA* ) and processed in automated equipment of the same brand. Samples were processed within two hours as recommended by the manufacturer.

Plasma Gal-3 levels were determined using an Enzyme-Linked Fluorescent Assay ( *ELFA* ) and measured on Biomerieux Vidas 30 ( *Biomerieux, Marcy l’Etoile, LY-France* ). Calibration of the assay was performed according to the manufacturer’s recommendations.

#### Echocardiography

The echocardiographic study was performed on a Sequoia 512 ultrasound device ( *Acuson, Mountain View, California, USA* ) with a 2.5 MHz transducer. All measurements were performed according to the American Society of Echocardiography Guidelines.^7^ A nasal respirometer was used for simultaneous recording of respiration, which was performed by an observer blinded to the other assessments of the protocol. Two-dimensional imaging was performed from parasternal, apical, and subcostal windows. The parasternal, apical views and M-mode recordings were used to detect the presence of respiratory ventricular septal motion. Apical views were also used to detect distortion of ventricular contours caused by constrictive pericardium. The subcostal view was used to identify diameters of the inferior vena cava. Doppler information was obtained from apical, subcostal, right supraclavicular, and parasternal imaging windows. From the apical window, pulsed-wave Doppler recordings at the level of the mitral leaflet tips were used to measure early (E) and atrial (A) diastolic velocities, deceleration time of the E wave, and respiratory variation in the E velocity. Tissue Doppler assessment of mitral annular motion was used to record and compare diastolic early (E) velocities at both the septal and lateral mitral annulus.

## Statistical analysis

Descriptive analysis was performed; for quantitative data, central tendency and dispersion measurements are reported as median and interquartile ranges.

Qualitative data was reported as frequencies and percentages. Gal-3 levels were compared between controls and case groups using Wilcoxon rank-sum test, and the chi-square test or Fisher's exact test was used for categorical data. Spearman correlation coefficient was used to analyze the association between Gal-3 levels, parameters of echocardiography, CMR and ergometric test. An alpha error < 5% was considered statistically significant, with a confidence interval of 95%.

Graphics and statistical analyses were performed with Microsoft Excel 2013 and Stata ( *version 13.0, Stata Corp., College Station, TX* ), with a 2-tailed p < 0.05 considered to be significant.

## Ethical issues

The institutional review board approved this study that is in compliance with the Declaration of Helsinki. The local ethics committee approved the protocol, and all participants signed a written informed consent.

## Results

### Baseline characteristics

Twenty-five patients with constrictive pericarditis underwent pericardiectomy.

Median age was 45 years (33-57) with a predominance of men (n = 19, 76%), and median body mass index (BMI) was 25.6 kg/m^2^. Comorbidities were hypertension, tobacco use, type 2 diabetes, and chronic arterial disease. All baseline characteristics are listed in Table 1 . In the control group, median age was 44 (33-53) years, and 19 were men.

**Table 1:** – Clinical and laboratory measures

Characteristic	Measure
Male sex, n (%)	19 (76)
Age years, median (IQR)	45 (33-57)
BMI, kg/m^2^, median (IQR)	25.6 (22-27)
Time of symptoms, months median (IQR)	24 (12-36)
Time of hospitalization, median (IQR)	8 (7-16)
Time of ICU after procedure, median (IQR)	2 (2-3)
**Comorbidities, n (%)**	
Hypertension	4 (16)
Type 2 diabetes	2 (8)
Chronic arterial disease	3 (12)
Tabagism	5 (20)
Atrial fibrillation	10 (40)
Low voltage ECG	6 (24)
Calcification X-ray	11 (44)
Pleural effusion X-ray	5 (20)
**NYHA functional class, n (%)**	
I	4 (16)
II	7 (28)
III	11 (44)
IV	3 (12)
**Clinical signs, n (%)**	
Jugular stasis	22 (88)
Edema	22 (88)
Ascites	18 (72)
Hepatomegaly	16 (64)
Pericardial knock	12 (48)
Kussmaul sign	6 (24)
Paradoxal pulse	5 (20)
**Laboratory measurements – median (IQR)**	
Galectin-3, ng/mL*	14.8 [9.7-17.2]
Hemoglobin, g/dL,	13.4 [12.8-14.3]
Creatinine, mg/dL,	1.02 [0.99-1.26]
CRP, mg/dL (range)	5.4 [3.2-9.4]
BNP, pg/mL (range)	143 [89-209]

Continuous variables are presented as median and interquartile range [IQR]. Categorical data are presented as percentage. *risk of postoperative death calculated by EuroSCORE (%). BMI: body mass index; ECG: electrocardiogram; ICU: intensive care unit; NYHA: New York Heart Association; BNP: B-type natriuretic peptide; CRP: C-reactive protein.

Regarding clinical characteristics, the median length of symptoms before admission was 24 (12-36) months. Median length of hospital and intensive care unit (ICU) stay was eight and two days, respectively. The main clinical signs in the patients were related to right heart failure – jugular vein distention (n = 22, 88%), edema (n = 22, 88%), ascites (n = 18, 72%) – and 16 (64%) had hepatomegaly (n = 16, 64%) at physical examination ( Table 1 ).

Fourteen (56%) patients presented with New York Heart Association (NYHA) functional class III/IV on admission. Analyzing the clinical signs, patients with ascites had higher Gal-3 levels (16.2 ng/mL [11.6-17.5]) compared with those without ascites, (8.2 ng/mL [6.6-14.8]), with no statistically significant difference though (p = 0.06; CI 95% 0.98-1.72). No association was found of Gal-3 levels with any of the other signs described.

The most frequent etiologic diagnosis was idiopathic (n = 19, 76%), tuberculosis (n = 3, 12%), followed by collagenases (n = 2, 8%), and post-surgery (n = 1, 4%). There were no deaths after pericardiectomy.

### Laboratory and complementary tests

The median values of hemoglobin, creatinine, C-reactive protein, BNP and Gal-3 are presented in Table 1 . Gal-3 levels were not significantly higher in CCP patients compared with control patients. The median level of Gal-3 was 14.8 [9.7-17.2] ng/mL and 11.8 [10.6-14.2] ng/mL for CCP and controls respectively (p = 0.22). In addition, no significant association was observed between Gal-3 levels and echocardiographic measures (left ventricular diastolic diameter, LVDD; left ventricular systolic diameter, LVSD, left atrial diameter, left ventricular ejection fraction), or with CMR measures (pulmonary artery systolic pressure, PASP > 55 mmHg; left atrial diameter; abnormal septal motion (septal bouncing); vena cava dilatation and myocardial and pericardial late enhancement).

### Imaging examinations

All subjects underwent echocardiography and CMR examinations. The median ejection fraction measured by echocardiography and CMR was 60% and 57%, respectively. Analyzing the echocardiographic parameters, only 13 (52%) of the results suggested constrictive pericarditis as a diagnosis. In addition, pericardial thickening was observed in 17 (68%) subjects and inspiratory variation in mitral and tricuspid flow was observed in 13 (52%), suggesting diastolic restriction ( Table 2 ).

**Table 2:** – Echocardiography and cardiac magnetic resonance variables

Echocardiography variables	N	%
Echo suggests CCP	13	52
Aortic sinus (mm)	30	(29-34)
Left atrium Diastolic diameter (mm)	43.5	(40-47)
Interventricular septum (mm)	8	(8-9)
Posterior wall (mm)	8	(8-9)
RVDD basal (mm)	28	(26-32)
LVDD (mm)	45	(41-46)
LVSD (mm)	29	(27-32)
LVEF (%)	60	(59-66)
Pericardial thickness (> 4 mm)	17	68
Respiratory flow variations (%)	13	52
**Cardiac magnetic resonance (CMR)**		
CMR suggests CCP	23	92
Pericardial enhancement	6	24
Miocardial enhancement	2	8
Septal bouncing	23	92
Increased Left atrium	22	88
Vena cava dilatation	23	92
LVEF (%)	57	(54-62)
Pericardial thickness (mm)	6	(5-8)
Pericardial thickness (> 4 mm)	21	84

*Continuous variables are presented as median and interquartile range [IQR], categorical data are presented as percentage. CCP: chronic constrictive pericarditis; RVDD: right ventricular diastolic diameter; LVDD: left ventricular diastolic diameter; LVSD: left ventricular systolic diameter; LVEF: left ventricular ejection fraction.*

The CMR images suggested a CCP diagnosis in 23 (92%) subjects. Pericardial thickness (> 4 mm) was found in 21 (84%) subjects, and the most frequent abnormalities observed were septal bouncing and aortic and vena cava dilatation, both observed in 23 (92%) patients, followed by increased left atrium in 22 (88%) ( Table 3 ).

**Table 3:** – Effect of pericardiectomy on functional capacity

Variables	Pre	Post	p
Velocity (mph)	2.5 [2-2.5]	3 [2.5-3.3]	0.001
Exercise time (min)	9.5 [6.9-11.7]	9.9 [4.5-14]	0.397
Peak HR (bpm)	139 [114-160]	159 [138-178]	0.020
VO_2_at AT (mL/kg/min)	13.5 [11.2-14.6]	16.4 [13.8-20.75]	0.002
AT (%)*	73 [60-81]	69.5 [62.5-78.5]	0.856
peak VO_2_(mL/kg/min)	18.5 [14.6-22.9]	25.4 [22.3-28.6]	< 0.001
peak VO_2_(%)*	63 [49.5-70.5]	82 [69.5-95]	< 0.001
Peak V_E_(L/min)	48 [41.3-57.6]	61.7 [44.5-79.9]	< 0.001
V_E_/VCO_2_slope RER	35.5 [30-40] 1.1	29 [28-31.5] 1.1	< 0.001 > 0.05

* Percentage in relation to predicted for age and sex. Continuous variables are presented as median and interquartile range [IQR], categorical data are presented as percentage. VO_2_: oxygen consumption; AT: anaerobic threshold; HR: heart rate; VO_2_: oxygen consumption; VE: pulmonary ventilation; RER: respiratory exchange ratio.

### Cardiopulmonary exercise test

In all subjects, the CPET proved to be safe, without serious complications. The tests were considered effective once median respiratory exchange ratio was 1.1 at both time points of the study. Overall, after surgical intervention, patients experienced improvement in cardiopulmonary capacity ( Table 3 ) in treadmill speed, peak heart rate, peak oxygen consumption (peak VO_2_) at anaerobic threshold (AT), AT, VO_2_and V_E_/VCO_2_slope.

BNP values had no correlation with CPET parameters either before or after the pericardiectomy procedure. However, although the Gal-3 marker did not correlate with CPET parameters before the procedure, we observed a moderate inverse correlation with test duration (r = –0.79; p < 0.001), exercise time (r = –0.79; p < 0.001), and heart rate at AT (r = 0.60; p = 0.01) in the postoperative period.

Histopathological study

Histological study was performed of 21 samples. Severe fibrosis and calcification were a common finding, observed in 19 (90.5%) and 12 (57.1%) of the specimens, respectively. Histopathological examination revealed mild inflammation in 16 cases (76.2%). No statistically significant association was found between Gal-3 and these findings ( Table 4 ).

**Table 4:** – Histopathologic analysis and galectin-3

	Mild	Severe
**Fibrosis**		
n (%)	2 (9.5)	19 (90.5)
Gal-3	19.7 [14.2-25.2]	14.8 [9.4-17.2]
**Calcification**		
n (%)	9 (42.9)	12 (57.1)
Gal-3	16.1 [11.9-20.3]	14.1 [9.5-16.2]
**Inflammation**		
n (%)	16 (76.2)	5 (23.8)
Gal-3	14.5 [10.5-16.9]	16.1 [9.3-25.2]

*Continuous variables are presented as median and interquartile range [IQR], categorical data are presented as percentage. No statistically significant difference was found between the groups.*

## Discussion

Our study showed that patients diagnosed with CCP had normal levels of Gal-3 preoperatively, comparable with the control group. Also, we observed no significant correlation of Gal-3 with echocardiographic and CMR measures and BNP. After pericardiectomy, we observed an improvement in peak VO_2_and V_E_/VCO_2_slope, which are makers of poor prognostic. However, we observed negative associations between Gal-3 and CPET parameters after pericardiectomy, such as test duration and exercise time.

Gal-3 has long been known to be a mediator of fibrosis in multiple organs, including the heart, kidney, pancreas, liver, and lung.^8^ Nevertheless, no studies have been performed on clinical data of CCP patients, associating levels of Gal-3 with pericardial structure, function, and functional status.

Ntsekhe et al.^5^ studied AcSDKP and galectin levels in normal pericardial fluid and tuberculous pericardial effusion.^5^ AcSDKP exerts part of its antifibrotic effect by inhibiting Gal-3, which is inactivated by angiotensin-converting enzyme (ACE). The authors concluded that depressed levels of AcSDKP in conjunction with normal or low Gal-3 levels within the pericardium may explain the high incidence of constrictive pericarditis associated with tuberculous pericarditis.

Constrictive pericarditis is a heterogeneous disorder and the risk of constriction after an acute episode is correlated with the etiology. Imazio et al.^9^ found a incidence of constrictive pericarditis < 0.5% in idiopathic or viral acute pericarditis; 2.8% for connective tissue disease, 4.0% for neoplastic pericarditis; 20% for tuberculous pericarditis, and 33% for purulent pericarditis. In our cohort, most patients (76%) had an idiopathic etiology, and we cannot extrapolate our results to other etiologies. Only a study with more patients and different etiologies may elucidate whether galectin can modulate constriction.

Inflammation is a physiological process that acts as a trigger for fibrosis and tissue regeneration following injury. The pericardium is a poorly vascularized structure with fibers composed of collagen that generally do not show the delayed enhancement observed with gadolinium injection. In cases where there is hyperemia and inflammation in the pericardium, there is increased vascularization resulting in an increase in late pericardial enhancement on CMR.^10^ Zurik et al.^2^ also observed that pericardial delayed hyperenhancement on CMR is common in patients with CCP and is associated with histological markers of chronic inflammation and increased neovascularization, which is indicative of an ongoing, dynamic active inflammatory reaction.^2^ Patients with CCP without pericardial delayed hyperenhancement had more pericardial fibrosis and calcification and a less pericardial thickening. We also did not observe any significant association of Gal-3 levels with pericardial thickness and pericardial late enhancement evaluated by CMR.

One explanation as to why some patients with CCP do not improve after surgery is myocardial atrophy after prolonged constriction, residual constriction, or a concomitant myocardial process that leads to prolonged cardiac failure in spite of successful pericardiectomy.^11 , 12^ Another possibility is myocardial fibrosis. Probably, the Gal-3-induced fibrosis is restricted to the myocardium and not the pericardium. We also did not observe an increase in galectin levels and myocardial delayed enhancement evaluated by CMR and histological analysis.

Cardiopulmonary testing is the most useful tool to objectively assess the exercise capacity of patients with systolic and diastolic heart failure.^6^ The test allows assessment of prognosis, efficacy of treatment, and selection for heart transplantation. Moreover, cardiopulmonary testing plays an important role in the prescription of exercises and rehabilitation programs.

Our patients improved peak VO_2_and V_E_/VCO_2_slope which are two independent predictors of mortality in heart failure patients with systolic or diastolic dysfunction.^13 , 14^ Also, an increase in peak VO_2_is associated with lower hospital readmissions in heart failure patients, demonstrating the importance of pericardiectomy for these patients.^15^

On the other hand, although numerous studies have evaluated the impact of pericardiectomy on the functional class of patients with CCP, most of them were retrospective case series.^10 , 12^ Moreover, clinical assessment based on NYHA classification is imprecise and subjective. Some patients do not experience recovery in their functional capacity and NYHA functional class.

The associations between Gal-3 levels and exercise intolerance after pericardiectomy suggest the possible role of Gal-3 in the pathophysiology of constrictive pericarditis. This hypothesis should be tested in longer follow-up studies. The finding of Gal-3 as a predictor of improvement in functional capacity is relevant, because it suggests that benefits of pericardiectomy appear to be lower in patients with higher levels of Gal-3.

## Conclusion

Gal-3 levels were normal in patients with CCP and did not correlate with morphological and functional measures. The associations between Gal-3 levels and exercise intolerance after pericardiectomy suggest the possible role of Gal-3 in the prognostic prediction after pericardiectomy.

### Limitations

The sample consisted of young patients with a predominance of idiopathic etiology in a tertiary cardiology center, which may represent selection bias and limit the external validity of the results. We had only a single time point measure of Gal-3, and therefore we did not assess dynamic changes in this biomarker over time.
